# Lésion Monteggia du groupe III association rare, à propos d’un cas

**DOI:** 10.11604/pamj.2017.27.208.9272

**Published:** 2017-07-20

**Authors:** Mohamed Amine Oukhouya, Hind Abou El Jaoud, Saad Andaloussi, Hicham Abdellaoui, Karima Atarraf, Lamia Chater, My Abderrahman Afifi

**Affiliations:** 1 CHU Hassan II Fès, Maroc

**Keywords:** Monteggia, Décollement épiphysaire, Enfant, Monteggia, epiphyseal separation, child

## Abstract

La lesion Monteggia du groupe III est une lésion très rare, elle survient généralement dans un contexte de traumatisme violent et passe souvent inaperçue. Nous rapportons le cas d’un garçon de 11 ans qui s’est présenté aux urgences pour un traumatisme fermé du membre supérieur, et chez qui le bilan radiologique a objectivé une fracture de l’olécrâne et décollement épiphysaire du radius avec luxation de la tête radiale, le patient a bénéficié d’un traitement orthopédique avec une bonne évolution après 3 mois de recul.

## Abstract (en)

Type III Monteggia lesion is very rare, usually occurring within a context of violent trauma and often going unnoticed. We report the case of a 11-year old boy presenting to the Emergency Department with blunt trauma of the upper limb. The radiological evaluation showed olecranon fracture and radial epiphyseal separation associated with dislocation of the radial head. The patient underwent orthopedic treatment with good outcome after a mean follow-up of 3 months.

## Introduction

Giovani batista Monteggia en 1814 a donné son nom à une lesion mixte atteignant le coude et l’avant-bras. Il s’agit d’une fracture de l’ulna associée à une luxation de la tête radiale, cette association parfois complexe peut intégrer le groupe III de la classification de Trillat. Leur traitement oppose les fractures récentes où il est simple et avant tout orthopédique aux fractures anciennes où le traitement devient difficile chirurgical et repose sur l’ostéotomie de l’ulna. Les auteurs se proposent à travers cette observation de faire une revue de littérature.

## Patient et observation

**Figure 1:**
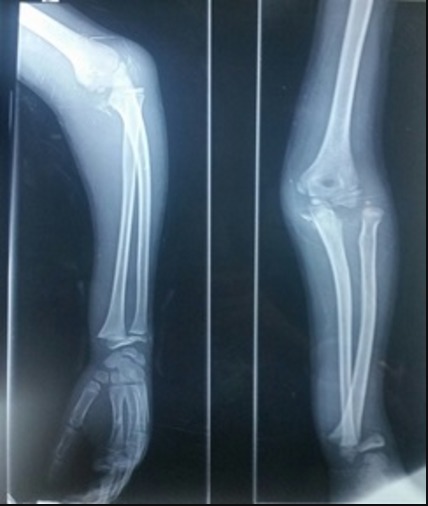
Radiographie de l’avant- bras: lésion Monteggia groupe III (Fracture de l’olécrâne+luxation de la tête radiale+décollement épiphysaire du radius)

**Figure 2:**
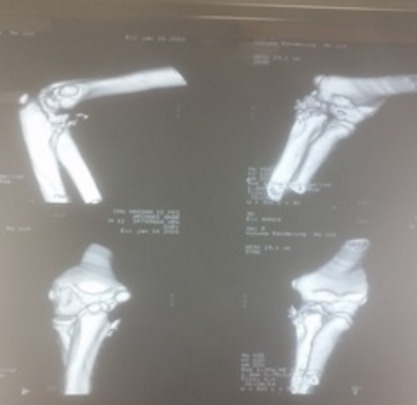
Scanner du coude (reconstruction 3D): fracture de l’olécrane avec détachement d’un fragment

**Figure 3:**
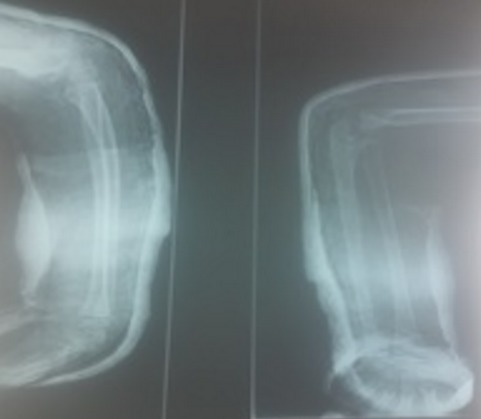
Radiographie de contrôle de l’avant-bras: réduction bonne

Il s’agit de A.M de sexe masculin agé de 11 ans, victime le jour de son admission d’une chute d’un Olivier avec reception sur la paume de la main coude en extension avant-bras en pronation occasionnant chez lui une douleur avec impotence fonctionnelle totale, chez qui l‘examen clinique trouve un coude déformé tuméfié douloureux et un poignet déformé en dos de fourchette tuméfié douloureux sans déficit vasculo-nerveux ni ouverture cutanée. La radiographie standard de l’avant-bras prenant le coude objective une lésion de monteggia associée à un décollement épiphysaire stade 2 du radius, association appartenant au groupe III de la classification de Trillat (Figure 1). Une réduction orthopédique de la luxation de la tête radiale et du décollement épiphysaire sous contrôle scopique a été réalisée avec mise en place d’une atelle postérieure avec manchette platrée. Le contrôle radiographique était satisfaisant (Figure 2 et Figure 3). L’évolution était bonne avec un recul de 3 mois.

## Discussion

La lésion Monteggia est rare chez l’enfant et lorsqu’elle est associée à un décollement epiphysaire distal des 2 os de l’avant-bras elle devient exceptionnelle [1, 2]. Selon la littérature un seul cas a été rapporté associant une lésion de Monteggia type 3 à un décollement épiphysaire stade 2 distal du radius entrant dans le cadre du groupe III [1]. Le mécanisme exact de la lésion reste encore mal élucidé, il est du à la transmission de manière ascendante d’une force rotatoire et varisante du poignet au coude alors que la fracture de l’extrémité distale du radius est due à une chute avec récéption sur la paume de la main poignet en dorsi-flexion et pronation de l’avant-bras [1, 3]. La manifestation clinique de ce type de lésion n’a rien de particulier à part une tuméfaction douloureuse du coude et du poignet. Le diagnostic est radiologique et reste souvent inaperçu par 50% des médecins aux urgences et par 25% des radiologues [1, 4], cette lésion est définie par une luxation de la tête radiale objéctivée par la rupture de la ligne de storen aussi bien de face que de profil associée à une fracture de l’ulna et à un décollement épiphysaire du radius [5, 6]. Le traitement de cette lésion chez l’enfant est essentiellement orthopédique ce qui était le cas pour notre patient, le recours à la chirurgie est rare et il n’est indiqué qu’en cas d’irréductibilité ou de réduction instable ou de récidive [5, 7, 8]. Pour notre cas on a eu recours au traitement orthopédique aussi bien pour la luxation de la tête radiale que pour le décollement épiphysaire du radius avec une stabilité qui était conservée.

## Conclusion

La lésion monteggia du groupe III est exceptionnelle. L’une des composantes de cette lésion peut passer inaperçue; ainsi une évaluation clinique appropriée, un examen radiologique et un traitement approprié sont la clé de la bonne évolution de ce type de lésion. Les auteurs ne déclarent aucun conflit d’intérêt.

## Conflits d’intérêts

Les auteurs ne déclarent aucun conflit d'interêt.
